# Large granular lymphocytic leukemia complicating autoimmune polyglandular syndrome type 1 in siblings

**DOI:** 10.1002/ccr3.1454

**Published:** 2018-03-11

**Authors:** Jonathan S. Harrison, Harsh Parmar, Xiangbing D. Wang

**Affiliations:** ^1^ Department of Medicine and the Carole and Ray Neag Cancer Center University of Connecticut School of Medicine Farmington Connecticut; ^2^ Department of Medicine Rutgers ‐ Robert Wood Johnson Medical School New Brunswick New Jersey

**Keywords:** Autoimmune polyglandular syndrome, large granular lymphocytic leukemia, pure red cell aplasia

## Abstract

Autoimmune polyglandular syndrome type 1 (APS1) is a rare autosomal recessive disorder, and large granular lymphocytic leukemia (LGLL) may, even more rarely, complicate APS1. LGLL may be subtle in presentation, but it is imperative to recognize LGLL in APS1 promptly, as outcome may otherwise be fatal, as described herein.

## Introduction

Large granular lymphocytic leukemia (LGLL), also known as T‐gamma lymphoproliferative disorder, is a clonal disorder of CD8‐positive T cells frequently complicated by cytopenias of a specific hematopoietic lineage. The cytopenias seen in LGLL may be isolated neutropenia, isolated thrombocytopenia, or pure red cell aplasia. Autoimmune polyglandular syndrome type 1 (APS type 1) is a rare, autosomal recessive disorder linked to mutations in the gene AIRE located on human chromosome 21. This disorder, also called autoimmune polyendocrinopathy–candidiasis–ectodermal dystrophy (APECED), most often presents with three major clinical components: recurrent mucocutaneous candidiasis, hypoparathyroidism, and adrenal insufficiency. AIRE encodes a 545 amino acid transcription factor, mutations in which are associated with loss of the normal deletion of T‐cell clones that, when persistent in an individual, mediate self‐immunity 1. Over one hundred mutations in the AIRE gene have been reported to date, and there is heterogeneity among patients with respect to frequent additional abnormalities seen in APS type 1. Minor components of the disease reported to date include recurrent fever with rash, retinal pigmentation, autoimmune hepatitis, alopecia, or vitiligo, among others 2. We encountered a woman with APS type 1 who, at age 23, developed pure red cell aplasia, and diagnostic evaluation documented LGLL with a clonal T‐cell population. One year after the proband patient developed pure red cell aplasia and LGLL, the proband's younger sister – who had then reached age 23 and who also had APS type 1 – developed clinically significant anemia. Diagnostic evaluation then documented LGLL with red cell hypoplasia in this younger sister. These observations suggest that the mutation in AIRE was causally related to development of LGLL and the consequent anemia with erythroid hypoplasia, in these two individuals.

## Case 1

**Figure 1:**
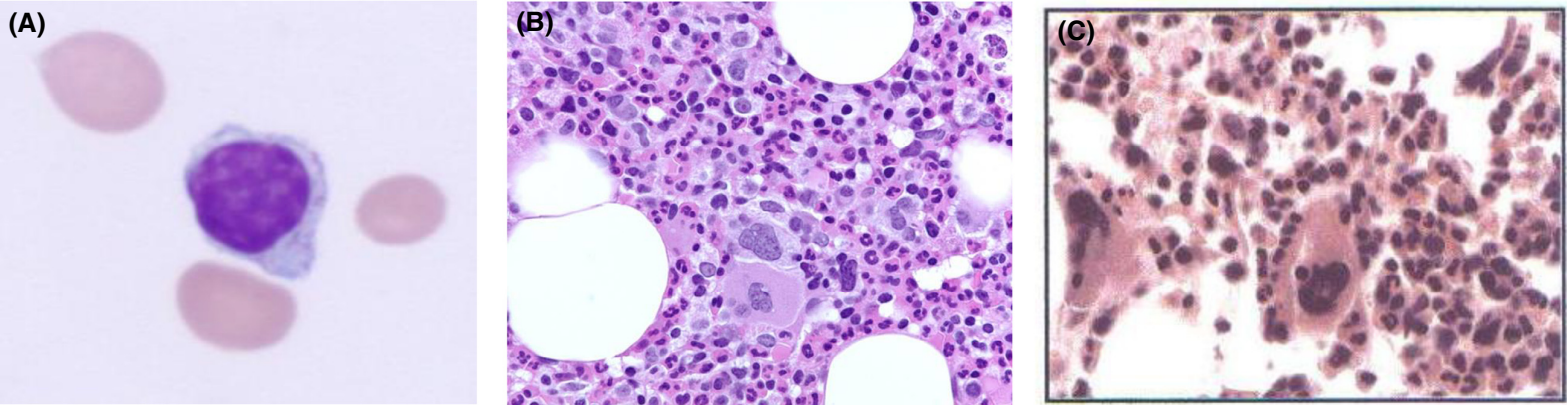
Blood count data from each case. (A) Large granular lymphocyte in the peripheral blood of proband (case 1). (B) Marrow biopsy showing red cell aplasia in proband (case 1). (C) Marrow biopsy showing red cell hypoplasia in sister of proband (case 2).

A 23‐year‐old woman presented to our medical center in August 2008 with complaints of progressive fatigue and exertional dyspnea of one‐month duration. At time of admission, hemoglobin was 6.5 gm/dL with normal leukocyte and platelet count. The patient's medical history was significant for autoimmune polyglandular syndrome type 1, identified during her teenage years. The patient had growth retardation noted by age 6, and developed clinically overt hypoparathyroidism at age 10. At age 14, adrenal insufficiency was documented; in addition, there was a history of recurrent oral candidiasis. Physical examination at initial presentation revealed a petite woman, awake, alert, and fully oriented in no acute distress, but reporting fatigue. Vital signs were normal aside from a resting tachycardia; the remainder of the examination was remarkable only for short stature and pale conjunctiva. Laboratory results at the time of hematology consultation included a total white blood cell count of 30.9 × 10^9^/L, hemoglobin of 6.6 g/dL (4.09 mmol/L), and platelet count of 181 × 10^9^/L. The mean corpuscular volume was 107.4 × 10^−15^L with reticulocyte count of 1.83%. Review of the peripheral blood film showed 87% segmented neutrophils, 5% lymphocytes, 3% monocytes, 2% eosinophils, 1% basophils, and 2% myelocytes. No further immature white blood cell forms were noted. The patient had received a “stress dose” of hydrocortisone on admission by the medical house staff, due to chronic corticosteroid replacement therapy and presumed acute illness. Total leukocyte counts fell toward normal range following reduction in corticosteroid dose (See Table 1). Schistocytosis was not observed in the peripheral blood. However, there were macrocytes, and occasional large granular lymphocytes were noted (Fig. 1A). Marrow examination showed pure red cell aplasia (Fig. 1B). Immunophenotyping of the blood and the marrow documented a clonal population of suppressor T cells, expressing CD2, CD3, CD5 (dim), CD7, CD8, and CD57. Molecular diagnostics by polymerase chain reaction amplification of the T‐cell receptor gamma chain (performed at the University of Washington Hematopathology Laboratory, Seattle, Washington) further documented a clonal T‐cell population in the blood and marrow. The patient was treated sequentially using high‐dose corticosteroids, followed by methotrexate, and subsequently cyclosporine A, all without significant improvement in erythropoiesis. She subsequently was admitted to hospital for further therapy, but died in November 2008, from what appeared to be infectious complications of immunosuppression. Autopsy documented intracranial hemorrhage, with a purulent infiltrate in the brain, and blood vessel findings are consistent with a vasculitis, but no bacterial or fungal organisms are identified.

**Table 1:** Clinical Parameters at times of diagnosis

Patient	Case 1 (Proband–older sister)	Case 2 (Younger sister)
Date of laboratory evaluation	12 August 2008	10 September 2009
White blood cell count (SI units: 10^9^/L)	17.5	9.8
Absolute lymphocyte count (SI units: 10^9^/L)	12.3	3.9
Hemoglobin (SI units: mmol/L)	4.03 (6.5 gm/dL)	6.14 (9.3 gm/dL)
Hematocrit (%)	19	28
Mean corpuscular volume (10^−15^L)	113	106
Reticulocyte count (%)	1.5	1.0
Platelet count (SI units: 10^9^/L)	181	226

Table of hematologic parameters for the two cases described. Data for Case 1 taken from the day of initial hospitalization; blood counts in the text of the manuscript are from the date of hematology consultation. Date for Case 2 is from the date of her outpatient bone marrow examination.

## Case 2

A 23‐year‐old woman, a younger sister of the proband, initially presented to an outside medical center with complaint of progressive fatigue of two‐month duration in September 2009. At that time, hemoglobin was 10.3 gm/dL with normal leukocyte and platelet count. The patient's medical history was significant for autoimmune polyglandular syndrome type 1; a diagnosis was made during her teenage years. The patient had growth retardation noted by age 6, and developed clinically overt hypoparathyroidism at age 12. At age 13, adrenal insufficiency was documented; in addition, there was a history of recurrent oral candidiasis in this younger sister. Upon identification of anemia in the year 2009 (see Table 1 for blood counts), given the history of her older sister having died the previous year, diagnostic evaluation was undertaken promptly. Review of the peripheral blood smear revealed large granular lymphocytes, and immunophenotyping of the blood confirmed the presence of a clonal population of T cells, expressing CD2, CD3 (dim), CD5 (dim), CD7, CD8, and CD57 (performed at Genoptix Medical Laboratory, Carlsbad, CA). Molecular diagnostic study of the marrow for the T‐cell receptor beta chain gene was rearranged from germ line (also performed at Genoptix Medical Laboratory), further confirming the presence of a clonal population of suppressor T cells. Marrow aspirate and biopsy demonstrated erythroid hypoplasia; interestingly, a small amount of reticulin fibrosis was also noted.

## Discussion

**Figure 2:**
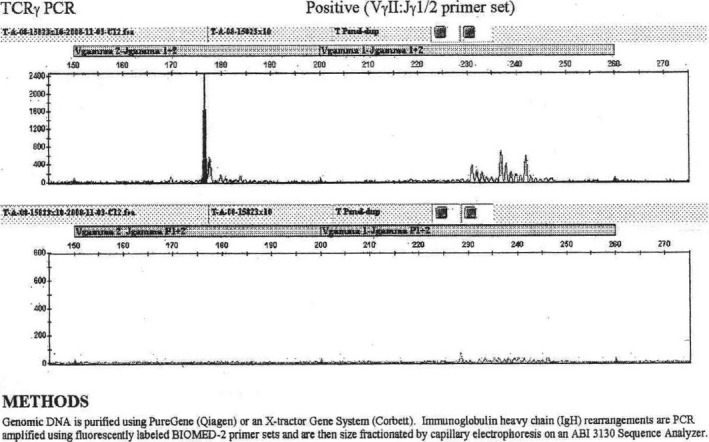
T‐cell clonality by polymerase chain reaction. Polymerase chain reaction amplification study documenting a clonal T‐cell population in the blood from the proband (see text).

Specimens of blood and marrow from both the proband with APS type 1 and her similarly affected younger sister each demonstrated a clonal population of CD8‐positive suppressor lymphocytes by immunophenotyping. The microscopic appearance of these cells was, as noted, classic for large granular lymphocytes (Fig. 1A). In both patients, the abnormal T cells marked positive for CD2, CD3, CD5 (dim), CD7 (dim) in one sibling and bright in the other, CD8 and CD57. The appearance of these cells was classic for large granular lymphocytes (Fig. 1). In both patients, analysis of the T‐cell receptor gene complex showed a clonal pattern, indicating a monoclonal population of T cells in each of the patients (Fig. 2). The T‐cell receptor *gamma*‐chain gene was analyzed in the proband by PCR, and the T‐cell receptor *beta* chain gene was analyzed by polymerase chain reaction in the second case, due to logistical constraints in the clinical care of the two patients at different centers. Taken together, these results establish that both siblings had, at discordant times, T‐gamma large granular lymphocytic leukemia complicating APS type 1. Marrow examination of the proband in the year 2008 documented pure red cell aplasia. Marrow examination of the second patient, the younger sister of the proband, documented erythroid hypoplasia that progressively worsened over subsequent months. This younger sister of the proband has been treated using oral methotrexate and recombinant erythropoietin, and remains alive but under active medical care approximately 8 years after diagnosis.

Large granular lymphocytic leukemia is an uncommon, clonal disorder of CD8‐positive suppressor T cells, which is typically an indolent disease. One prominent hypothesis regarding the etiology of this disorder is that chronic antigenic stimulation results in the polyclonal expansion of T cells, which includes a clone that eventually becomes dominant, leading to a monoclonal population. If this clone recognizes a self‐antigen, then, due to regulatory failure and lack of deletion of that T cell clone, the disorder results in clinical symptomatology on an autoimmune basis. In the case of this particular family, the consequence of autoimmune polyglandular syndrome was not simply autoimmune polyendocrine failure, but the development of CD8‐positive large granular lymphocytic leukemia, also termed T‐gamma disease, and categorized by the World Health Organization as a form of mature T‐cell lymphoma. In both of these cases, the patient's clone of large granular lymphocytes appears to have targeted an erythroid antigen, resulting in erythroid hypoplasia and clinically significant anemia, suggesting that a similar T‐cell receptor rearrangement may have been present in both siblings. Unfortunately, sequencing of the T‐cell receptor genes in these patients was not logistically possible, and thus, the sequences could not be compared.

There are a small number of case reports and small series of patients with APS1 developing large granular lymphocytic leukemia complicated, in turn, by pure red cell aplasia. A review of the literature reveals six cases of APS1 associated with pure red cell aplasia prior to the present report. Orlova and colleagues recently reviewed this literature, following their experience with a 26‐year‐old woman with APS1 who developed pure red cell aplasia responsive to mycophenolic acid 3. Prior reports of pure red cell aplasia in the setting of APS1 have not described siblings with pure red cell aplasia complicating the course of more than one family member 4, 5, 6. Of the cases reported to date, no siblings have been described previously. Familial LGLL has been reported, but only in a father and son without evidence of APS1 7.

## Conclusion

It may not be surprising that LGLL and pure red cell aplasia developed in both of the sisters described herein, as these siblings must, of course, have shared the same mutation in the AIRE gene. The diagnosis of LGLL complicated by pure red cell aplasia in the proband accelerated the recognition of the disease in the younger sister and may thus have improved the outcome for the second patient. Thus, it is important that, in patients with APS1, these individuals be closely monitored for the development of cytopenias. If cytopenias do occur, then the possibility of LGLL in the differential diagnosis should be considered early, as this may result in an improved outcome for the patient 8.

## Authorship

JSH: participated in care of both patients. JSH and XDW: participated in care of the proband patient. JSH and XDW: authored the manuscript. JSH, HP and XDW: critically reviewed and edited the manuscript and approved the final manuscript.

## Conflict of Interest

None of the authors has existing conflict of interests to report.
